# Sub-Classification of Lateral Cervical Lymph Node Metastasis in Papillary Thyroid Carcinoma by Pathologic Criteria

**DOI:** 10.1371/journal.pone.0133625

**Published:** 2015-07-17

**Authors:** Min Ji Jeon, Won Gu Kim, Eun Kyung Jang, Yun Mi Choi, Dong Eun Song, Tae-Yon Sung, Jong Ho Yoon, Ki-Wook Chung, Suck Joon Hong, Jin-Sook Ryu, Ji Min Han, Tae Yong Kim, Young Kee Shong, Won Bae Kim

**Affiliations:** 1 Department of Internal Medicine, Asan Medical Center, University of Ulsan College of Medicine, Seoul, Korea; 2 Department of Pathology, Asan Medical Center, University of Ulsan College of Medicine, Seoul, Korea; 3 Department of Surgery, Asan Medical Center, University of Ulsan College of Medicine, Seoul, Korea; 4 Department of Nuclear Medicine, Asan Medical Center, University of Ulsan College of Medicine, Seoul, Korea; 5 Department of Medicine, Samsung Changwon Hospital, Sungkyunkwan University School of Medicine, Changwon, Korea; University of Campinas, BRAZIL

## Abstract

**Background:**

Lateral cervical lymph node (LCLN) metastasis, or pathologic N1b disease, is an important risk factor in papillary thyroid carcinoma (PTC). However, many patients have favorable prognosis even with pathologic N1b patients in clinical practice. The study aims to identify high- and intermediate-risk groups based on initial pathologic characteristics in these patients.

**Patients:**

This study included 518 classical PTC patients confirmed as pathologic N1b at initial surgery between 2001 and 2010. All patients underwent a single fixed activity (5.6 GBq) of radioactive I-131 remnant ablation.

**Results:**

Patients with a primary tumor larger than 4 cm, gross extrathyroidal extension, metastatic LN larger than 3 cm, or greater than 10 metastatic LCLN were classified as high-risk group. These comprehensive pathologic criteria were retrieved from cox proportional hazard models. Twenty two percent of patients (n = 113) were classified as high-risk and 78% (n = 405) as intermediate-risk group. Successful ablation was identified in only 32% of the patients in the high-risk group and 61% in the intermediate-risk group (*p* < 0.001). The difference between the two risk groups was independent to gender. There was a significant difference in disease-free survival between the high- and intermediate- risk N1b groups during 5.1 years of median follow-up (84% vs. 59%, *p* < 0.001). Distant metastasis was more prevalent in the high-risk group (20%) than in the intermediate-risk group (4%, *p* < 0.001).

**Conclusions:**

The prognosis of PTC patients with LCLN metastasis varies depending on initial pathologic characteristics. We proposed the comprehensive pathologic criteria for sub-classification of N1b into high- and intermediate-risk groups and this sub-classification may permit personalized management of N1b PTC patients.

## Introduction

Lateral cervical lymph node (LN) involvement in papillary thyroid carcinoma (PTC), or pathologic N1b disease according to the traditional American Joint Cancer Committee/Union Internationale Contre le Cancer (AJCC/UICC) Tumor Node Metastasis (TNM) staging system, is an important risk factor for recurrence, distant metastasis and cancer-specific death [1–5]. Lateral cervical LN (LCLN) metastasis of PTC occurs in 10–24% of patients and the prevalence of recurrent/persistent disease in patients with pathologic N1b disease is greater than 30% [1, 5–8]. PTC patients with pathologic N1b disease typically require aggressive management, including high-dose radioactive iodine (RAI) remnant ablation [6, 9].

Given the increased prevalence of small, early-stage PTCs [10], the disease extent of pathologic N1b disease seems to be decreasing and many patients with pathologic N1b PTC have favorable prognosis than we expected in clinical practice. Therefore, it is important to distinguish higher risk from lower risk patients to optimize the management of PTC patients with pathologic N1b disease. Previous studies suggested several pathologic characteristics for risk stratification of pathologic N1b disease in PTC. Large metastatic foci in cervical LNs [11, 12], high numbers of metastatic LNs [13, 14], high numbers of metastatic LCLNs [15] and bilateral LCLN metastasis [16] have been identified as poor prognostic factors associated with recurrence or distant metastasis in PTC with pathologic N1b disease. However, no comprehensive criteria exist for optimal risk assessment of PTC with LCLN metastasis.

In this study, we aim to identify the comprehensive criteria for PTC patients with pathologic N1b according to initial clinicopathologic characteristics. Our criteria adopted several clinicopathologic prognostic factors from previous studies and we validated prognostic values of these factors in our patients. We analyzed whether sub-classification of N1b using this criteria may effectively predict the risk of structural recurrent/persistent disease during follow-up.

## Materials and Methods

### Study cohort

A total of 4,297 patients with classical PTC underwent total thyroidectomy at Asan Medical Center (Seoul, Korea) between 2001 and 2010. Among this cohort, 570 patients with pathologic N1b disease were identified and their medical records were reviewed. We excluded patients in whom distant metastases were found before initial surgery (n = 10) and patients who missed follow-up after initial treatment (n = 42). Finally, 518 patients were eligible for analysis. All patients also underwent therapeutic central and lateral neck dissection and subsequent RAI remnant ablation therapy with a single fixed activity of 5.6 GBq I-131 [17]. This study was approved by the institutional review board of the Asan Medical Center.

### Follow-up protocol

After initial therapy, patients took levothyroxine for TSH suppression and were regularly followed-up with physical examination. Neck ultrasonography (US) and measurement of the levels of serum thyroglobulin (Tg) were done every 6 to 12 months with serum anti-Tg antibody (TgAb) [18]. Diagnostic radioiodine whole body scan (WBS) with measurement of serum stimulated Tg (sTg) level were done during the first one year after initial therapy. Additional diagnostic imaging studies, such as computed tomography, magnetic resonance scan or whole body fluoro-deoxyglucose (FDG)—positron emission tomography (PET) scan, were also performed in some patients as needed [17, 19].

### Definitions

Successful ablation was defined as negative serum sTg level (< 1 ng/mL) with negative TgAb (< 60 ng/ mL) and absence of suspicious metastatic lesions by neck US at the first follow-up after initial therapy. In patients with positive TgAb (≥ 60 IU/mL), successful ablation was defined as negative findings by neck US and diagnostic WBS. These definitions were from ESTIMABL study [20].

Clinical outcome was also assessed at the end of the follow-up period. Structural recurrent/persistent disease was defined as cytologically or histopathologically confirmed metastatic lesions and/or metastatic lesions in other distant organs with definite malignant features in various imaging studies and elevated serum Tg value [17].

### Statistics

R version 3.03 and R libraries survival, car, ggplot2, Cairo, rms, grid, plyr, Formula and lattice were used to analyze data and draw graphs (R Foundation for Statistical Computing, Vienna, Austria, http://www.R-project.org).

Continuous variables were presented as medians with inter-quartile range (IQR) and categorical variables as numbers with percentages. Structural recurrent/persistent disease was primary endpoint and Cox proportional hazard model was used to evaluate the risk of recurrence/persistence of disease. The proportion of variation in survival time explained (PVE) by the maximum likelihood ratio (G^2^) from Cox model (PVE = 1-exp (-G^2^/n)) was used to determine the most reliable criteria for sub-classification of N1b PTC patients. The PVE (%) ranges from 0 to 100 and the higher value suggests the more accurate predictability of risk of recurrence [17]. The internal stability of the comprehensive criteria was tested using bootstrap resampling [21, 22]. We created new data set by 200 random sampling of the original data and calculated the new Cox regression. We also calculated the cox regression and the PVE values using continuous variables [23]. Wilcoxon rank-sum test, Chi-square test, and Fisher’s exact test were used to compare the clinico-pathologic variables of patients according to the risk stratification. Disease-free survival curves based on risk were constructed with the Kaplan-Meier method and the log rank test was used to compare the curves. All *P* values were two sided, with *P* < 0.05 considered statistically significant.

## Results

### Prognostic factors for predicting structural persistent/recurrent disease of N1b PTC patients

A total of 518 patients were included in this study. The baseline characteristics of patients were described in Table 1. First, we identified the prognostic factors for predicting structural persistent/recurrent disease of N1 PTC patients in our patients. Patients were classified according to their age at initial surgery (≥ 45 years) [2, 4], gender [24], primary tumor size (> 4 cm) [25, 26], presence of gross extrathyroidal extension [25, 27], maximal size of metastatic LNs (≥ 3 cm) [12, 27], number of metastatic LNs (> 20) [13], number of metastatic LNs in only lateral neck (>10) [15], or presence of bilateral lateral cervical involvement [16] based on previous studies. In the univariate analysis of Cox proportional hazard model, all except the old age were significant prognostic factors. The results of univariate analysis using continuous variables were consistent with the results using categorical variables (Table 1).

**Table 1:** Univariate analysis of prognostic factors of persistent/recurrent disease in N1b PTC patients.

Characteristics	N (%)	Median (IQR)	Hazard ratio (95% CI)		*P*-value
Age		45 (37–54)	1.01	(0.99–1.03)	0.128
< 45 years	248 (48)		Ref		
≥ 45 years	270 (52)		1.26	(0.87–1.82)	0.229
Gender					
Female	372 (72)		Ref		
Male	147 (28)		2.00	(1.37–2.91)	< 0.001
Primary tumor size		1.6 (1.2–2.6)	1.24	(1.14–1.35)	< 0.001
≤ 4 cm	482 (93)		Ref		
> 4 cm	36 (7)		2.89	(1.74–4.79)	<0.001
Gross extrathyroidal extension					
No	504 (97)		Ref		
Yes	14 (3)		5.03	(2.54–9.96)	<0.001
Maximal metastatic LN size		1.1 (0.7–1.6)	1.49	(1.28–1.74)	< 0.001
< 3 cm	498 (96)		Ref		
≥ 3 cm	20 (4)		3.04	(1.63–5.66)	<0.001
No. of involved LNs		9 (5–13)	1.05	(1.03–1.07)	< 0.001
≤ 20	469 (91)		Ref		
> 20	49 (9)		2.09	(1.28–3.42)	0.003
No. of involved LNs in lateral neck		5 (3–8)	1.07	(1.04–1.10)	< 0.001
≤ 10	447 (86)		Ref		
> 10	71 (14)		2.55	(1.78–3.87)	< 0.001
Bilateral cervical involvement					
No	470 (91)		Ref		
Yes	48 (9)		2.08	(1.25–3.44)	0.005

In multivariate analysis, male gender, primary tumor larger than 4 cm, presence of gross extrathyroidal extension, more than 10 metastatic LCLNs, and larger metastatic LNs equal or greater than 3 cm were significant prognostic factors (Table 2). The number of metastatic LNs in lateral neck was a more evident prognostic factor compared to the number of metastatic LNs in the whole cervical compartments. In the Cox regression model using continuous variables, primary tumor size, gross extrathyroidal extension and maximal size of metastatic LNs were significant prognostic factors (Table 3).

**Table 2:** Multivariate analysis of prognostic factors of persistent/recurrent disease in N1b PTC patients.

Characteristics	#1			#2		
	Hazard ratio (95% CI)		*P*-value	Hazard ratio (95% CI)		*P*-value
Gender						
Female	Ref			Ref		
Male	1.56	(1.07–2.37)	0.023	1.59	(1.07–2.36)	0.023
Primary tumor size						
≤ 4 cm	Ref			Ref		
> 4 cm	1.87	(1.07–3.28)	0.028	1.79	(1.03–3.14)	0.040
Gross extrathyroidal extension						
No	Ref			Ref		
Yes	3.49	(1.66–7.33)	<0.001	3.01	(1.41–6.42)	0.004
Maximal metastatic LN size						
< 3 cm	Ref			Ref		
≥ 3 cm	2.60	(1.38–4.87)	0.003	2.57	(1.37–4.82)	0.003
No. of involved LNs						
≤ 20	Ref					
> 20	1.35	(0.73–2.47)	0.338			
No. of involved LNs in lateral neck						
≤ 10				Ref		
> 10				1.74	(1.05–2.88)	0.032
Bilateral cervical involvement						
No	Ref			Ref		
Yes	1.46	(0.78–2.71)	0.236	1.24	(0.67–2.28)	0.492

**Table 3:** Multivariate analysis of continuous prognostic factors of persistent/recurrent disease in N1b PTC patients.

Characterisitics	#1			#2		
	Hazard ratio (95% CI)		*P*-value	Hazard ratio (95% CI)		*P*-value
Gender						
Male	1.37	(0.91–2.08)	0.135	1.40	(0.92–2.12)	0.113
Primary tumor size	1.14	(1.03–1.26)	0.012	1.13	(1.02–1.26)	0.002
Gross extrathyroidal extension						
Yes	3.03	(1.44–6.39)	0.003	3.00	(1.43–6.30)	0.004
Maximal metastatic LN size	1.38	(1.17–1.63)	<0.001	1.37	(1.16–1.62)	<0.001
No. of involved LNs	1.02	(0.99–1.05)	0.104			
No. of involved LNs in lateral neck				1.03	(0.99–1.06)	0.150
Bilateral cervical involvement						
Yes	1.27	(0.71–2.26)	0.424	1.31	(0.73–2.32)	0.364

### New comprehensive criteria for sub-classification of N1b PTC patients

We made four different criteria for sub-classification of N1b PTC patients based on the result of the cox analysis. Model 1 defined the high risk group as male patients or patients with a primary tumor > 4 cm, gross extrathyroidal extension, maximal metastatic LNs ≥ 3 cm, over 10 metastatic LCLNs or bilateral cervical involvement. Others were classified into the intermediate risk group. Model 2 used prognostic factors as Model 1 except gender; patients with a primary tumor > 4 cm, gross extrathyroidal extension, metastatic LNs ≥ 3 cm, over 10 metastatic LCLNs, or bilateral cervical involvement were defined as the high risk group. Model 3 defined the high risk group as male patients or patients with a primary tumor > 4 cm, gross extrathyroidal extension, maximal metastatic LNs ≥ 3 cm or over 10 metastatic LCLNs. Model 4 used prognostic factors as Model 3 except gender; patients with a primary tumor > 4 cm, gross extrathyroidal extension, metastatic LNs ≥ 3 cm, or over 10 metastatic LCLNs were defined as the high risk group. When we compare the PVE value of the risk models, all comprehensive risk models had higher PVE values than each prognostic factor. Especially, Model 4 presented the highest value and we used Model 4 as the new comprehensive criteria for sub-classification of N1b PTC patients. The results of internal stability test were consistent with the results from original data set (Tables 4 and 5).

**Table 4:** Comparison of PVE values of prognostic factors.

Characterisitcs	PVE (%)
Gender	
Male	2.5
Primary tumor size	4.0
> 4 cm	2.7
Gross extrathyroidal extension	
Yes	2.9
Maximal size of metastatic LNs	4.1
≥ 3 cm	1.9
No. of involved LNs in lateral neck	3.8
> 10	3.4
Bilateral cervical involvement	
Yes	1.4

**Table 5:** Comparison of the risk models using PVE values with internal stability testing.

Models	Original PVE (%)	PVE (%) from bootstrap resampling
Model 1	5.3	5.2
Model 2	6.2	5.9
Model 3	4.9	4.5
Model 4	6.3	6.1

### Clinicopathological features according to the sub-classification

**Fig 1:**
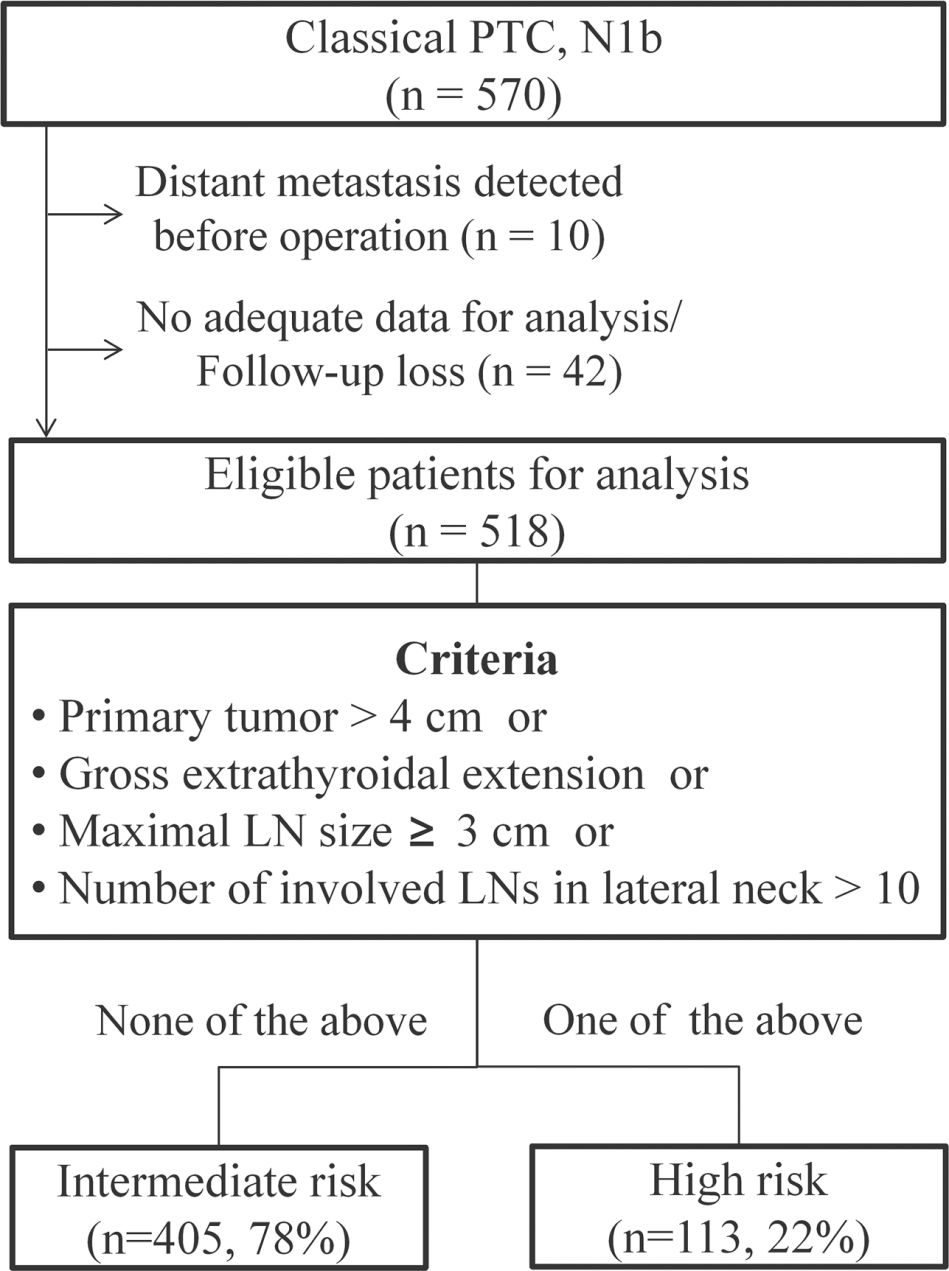
Sub-classification of N1b PTC patients. Of 518 patients who were eligible for this study, 113 (22%) patients were classified as high- risk based on pathologic characteristics.

Of 518 eligible patients with N1b disease, 405 (78%) were classified as intermediate-risk and 113 (22%) as high-risk according to Model 4 (Fig 1). The median age of patients at initial surgery was similar between the two risk groups (*P* = 0.546). Age distribution was also not associated with risk (*P* = 0.733); however, the gender distribution was different between the groups (*P* < 0.001). About half of the high-risk patients (44%), but only 24% of the intermediate-risk patients were male. By definition, patients in the high-risk group presented with larger primary tumors, more metastatic LCLNs, and larger metastatic LNs than those in the intermediate-risk group. High-risk patient also had a higher number of LNs resected and a higher LN ratio (*P* < 0.001) (Table 6).

**Table 6:** Clinicopathological features according to the sub-classification.

Characteristics	Intermediate risk (n = 405)	High risk (n = 113)	*P*-value
Age at diagnosis (yr)	45 (37–53)	46 (39–57)	0.546
≥ 45	209 (52)	61 (54)	0.733
Sex			
Male	96 (24)	50 (44)	<0.001
Size of primary tumor (cm)	1.5 (1.0–2.3)	2.7 (1.6–5.0)	<0.001
≤ 1	103 (25)	13 (12)	N/A
1.1–2	183 (45)	33 (29)	
2.1–4	119 (29)	31 (27)	
> 4	N/A	36 (32)	
Number of metastatic LNs	8 (5–12)	17 (11–23)	<0.001
Number LNs resected	38 (30–49)	53 (35–67)	<0.001
LN ratio	0.14 (0.08–0.22)	0.26 (0.20–0.38)	<0.001
Maximal size of metastatic LNs (cm)	1.0 (0.6–1.5)	1.5 (1.0–2.5)	<0.001
< 1	174 (43)	26 (23)	N/A
1~2.9	231 (57)	67 (59)	
≥ 3	N/A	20 (18)	
Number of metastatic LNs in lateral neck	4 (2–6)	12 (6–15)	<0.001
≤ 5	277 (68)	26 (23)	N/A
6~10	128 (32)	16 (14)	
> 10	N/A	71 (63)	
Bilateral cervical LN involvement			
Yes	12 (3)	36 (32)	<0.001

### Success rate of RAI remnant ablation

**Fig 2:**
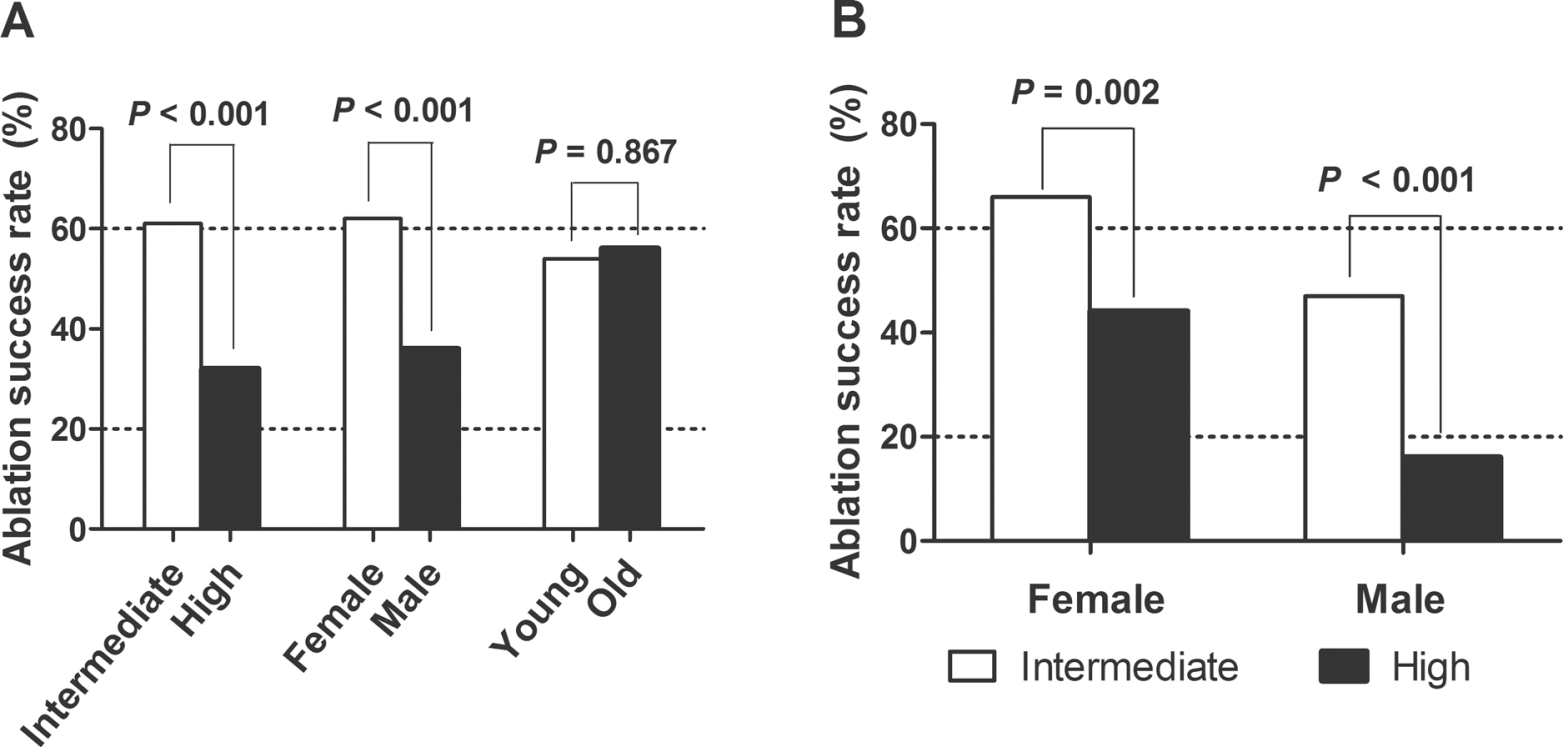
Ablation success rate of N1b PTC patients. (A) Ablation success rate based on the risk group, gender, and age. Our sub-classification and gender was significantly correlated with successful ablation rate 1 year after initial therapy. (B) Ablation success rate based on the risk group in both gender subgroups. The white bar indicates the intermediate-risk group and the black bar indicates the high-risk group. High-risk patients had a significantly lower ablation success rate than intermediate-risk patients in all subgroups.

We analyzed the success rate of RAI remnant ablation based on the risk group, gender, and age (Fig 2A). The ablation success rate was 61% in the intermediate-risk group but, only 32% in the high-risk group (*P* < 0.001). It was significantly lower in males (36%) than in females (62%, *P* < 0.001); however, there was no significant difference between the two age groups (54% in < 45 years vs. 56% in ≥ 45 years, *P* = 0.867). We analyzed the ablation success rate based on gender and the risk group (Fig 2B). The high-risk group had a significantly lower ablation success rate than the intermediate- risk group in both gender subgroups. High-risk males had the lowest ablation success rate as 16%.

### Long-term clinical outcome

**Fig 3:**
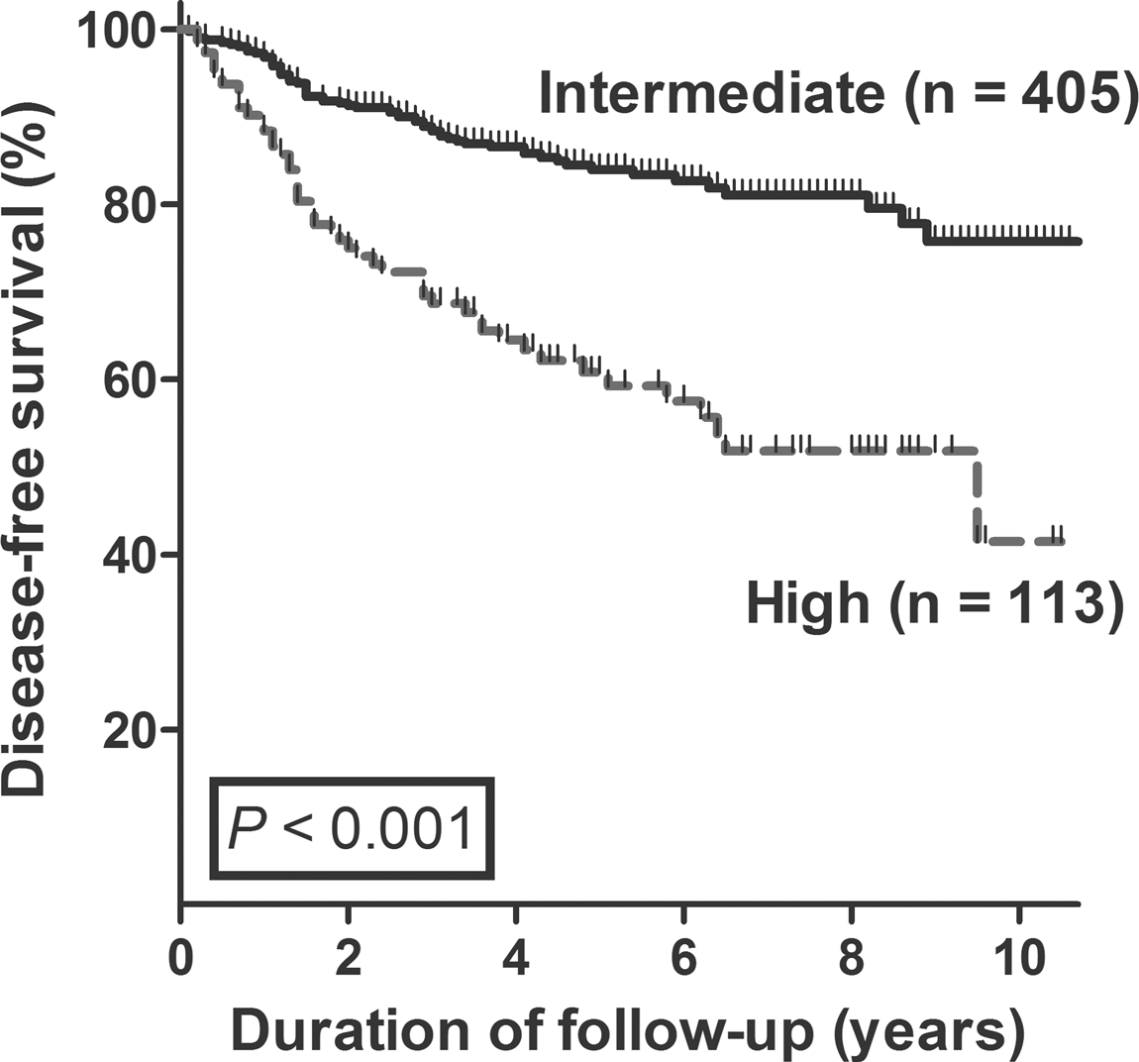
Disease-free survival based on sub-classification of N1b PTC patients. Patients in the high-risk group had significantly lower disease-free survival than patients in the intermediate-risk group.

The median duration of follow-up was 5.1 years (IQR 3.8–7.5). At the end of follow-up, 114 patients (22%) had structural persistent/recurrent disease, and 39 patients among them (8%) had distant metastatic disease. Significant differences in long-term clinical outcome were observed between the high- and intermediate-risk groups. The disease-free survival rate during the median follow-up period was 84% in the intermediate-risk group and 59% in the high-risk group (*P* < 0.001; Fig 3). Twenty three patients (20%) in the high-risk group and 16 (4%) in the intermediate-risk group developed distant metastatic disease during follow-up (*P* < 0.001). Disease- specific mortality occurred in only one patient in the high-risk group during follow-up.

## Discussion

In the present study, we propose the comprehensive pathologic criteria using primary tumor size, presence of gross extrathyroidal extension, maximal size of metastatic LNs, and the number of metastatic LNs in lateral neck for sub-classification of patients with N1b disease. We selected these pathologic characteristics as prognostic factors based on previous studies and validated them in our study patients. In multivariate analysis, larger primary tumor (> 4 cm), presence of gross extrathyroidal extension, larger metastatic LNs (≥ 3 cm) and more metastatic LCLNs (> 10) were significant prognostic factors predicting recurrent/persistent disease of PTC. Although male gender was also a significant prognostic factor in the multivariate analysis, inclusion of gender as a prognostic factor in the criteria decreased the PVE values. Therefore, we defined the four pathologic characteristics of N1b PTC as comprehensive pathologic criteria for sub-classification of N1b PTC.

This study suggested sub-classification of N1b PTC because the clinical outcome of N1b disease is variable. In the high- risk group, ablation success was achieved in only 32% of patients 1 year after initial surgery and high activity (5.6 GBq) RAI ablation. However, ablation success was confirmed in 61% of the patients and 84% of them remained disease-free during follow-up in the intermediate-risk group. They had a more favorable prognosis than we expected and accounted for about 80% of all N1b patients. These results are comparable to the prognosis of patients with only central cervical LN involvement (N1a) of PTC from previous reports [1, 19]. These findings suggest that there is heterogeneity among PTC patients with LCLN metastasis and the accurate risk stratification for these patients is needed. Our sub-classification criteria could be easily applicable in the clinical practice and will help physicians tailor the management of PTC patients with pathologic N1b disease.

Thyroid cancer is the only malignancy in which the age of patients is a prognostic indicator in the major staging systems [2, 28]. It is known that recurrence and mortality are more frequent in older patients than younger patients [3, 7, 11, 29, 30]. Low RAI responsiveness, high TSH level and impaired immune system in aging may be associated with poorer prognosis [28]. However, a relationship between advanced age and poor prognosis was not always observed. In this study, the ablation success rate was not different between the age groups. The proportion of patients older than 60 years was 15% in our patients, which is significantly lower than the previous study, even though the median age was similar [4]. This could be the reason for the discrepancy between ours and previous studies.

Interestingly, ablation success rate was significantly different between males and females in this study. Male N1b patients had significantly poorer prognosis than female. Males tended to have poorer prognostic pathologic characteristics and about half of the patients in the high-risk group were male in the present study. The European organization for research and treatment of cancer (EORTC) scoring system[31] and the AMES (Age, Grade, Extent, and Size) system [32] also consider gender as a prognostic factor and males seem to have more recurrences than females according to previous studies [29, 33, 34]. However, it is still unclear whether male gender should be used as a criterion of higher risk N1b.

This study has an inherent limitation in its retrospective design. We could not analyze the impact of extranodal extension of LN metastasis and maximal size of metastatic LNs in the lateral cervical compartment because our pathology data were insufficient. We also could not evaluate the impact of our sub-classification on cancer-specific survival because of a relatively short follow-up period. As well, our sub-classification is only useful for estimating the clinical outcome of patients who underwent total thyroidectomy with therapeutic central and lateral neck dissection and subsequent RAI remnant ablation therapy. Nevertheless, our study is unique since all eligible patients, representing a large patient population, were treated with a single fixed high-dose of RAI for remnant ablation which facilitates the comparison of clinical outcomes between the risk groups. In addition, our study subjects were managed at a single large center with uniform strategies, which minimizes confounding factors.

In conclusion, patients with N1b disease in PTC have variable outcomes according to the initial pathologic characteristics. Sub-classification of N1b into high- and intermediate-risk groups according to primary tumor size, presence of gross extrathyroidal extension, maximal size of metastatic LNs, and number of metastatic LCLNs could predict ablation success rate, structural recurrent/persistent disease, and distant metastasis. These findings suggest that sub-classification of N1b could be useful for personalized management of patients.

## References

[1] de Meer SG, Dauwan M, de Keizer B, Valk GD, Borel Rinkes IH, Vriens MR. Not the number but the location of lymph nodes matters for recurrence rate and disease-free survival in patients with differentiated thyroid cancer. World J Surg. 2012;36(6):1262–7. 10.1007/s00268-012-1427-1. 10.1007/s00268-012-1427-1 22270993 PMC3348473

[2] Greene FL, Page DL, Fleming ID, Balch CM, Fritz AG. AJCC cancer staging handbook: from the AJCC cancer staging manual: Springer Verlag; 2002.

[3] Smith VA, Sessions RB, Lentsch EJ. Cervical lymph node metastasis and papillary thyroid carcinoma: does the compartment involved affect survival? Experience from the SEER database. J Surg Oncol. 2012;106(4):357–62. 10.1002/jso.23090. 10.1002/jso.23090 22392921

[4] Verburg FA, Mader U, Tanase K, Thies ED, Diessl S, Buck AK, et al Life expectancy is reduced in differentiated thyroid cancer patients ≥ 45 years old with extensive local tumor invasion, lateral lymph node, or distant metastases at diagnosis and normal in all other DTC patients. J Clin Endocrinol Metab. 2013;98(1):172–80. 10.1210/jc.2012-2458 23150687

[5] Jeon MJ, Kim TY, Kim WG, Han JM, Jang EK, Choi YM, et al Differentiating the location of cervical lymph node metastasis is very useful for estimating the risk of distant metastases in papillary thyroid carcinoma. Clinical endocrinology. 2014;81(4):593–9. Epub 2014/04/23. 10.1111/cen.12463 . 24750108

[6] Sabra M, Grewal R, Ghossein R, Tuttle RM. Higher administered activities of radioactive iodine are associated with less structural persistent response in older, but not younger, papillary thyroid cancer patients with lateral neck lymph node metastases. Thyroid. 2014 10.1089/thy.2013.0465. . 24559250

[7] Nixon IJ, Ganly I, Patel SG, Palmer FL, Di Lorenzo MM, Grewal RK, et al The results of selective use of radioactive iodine on survival and on recurrence in the management of papillary thyroid cancer, based on Memorial Sloan-Kettering Cancer Center risk group stratification. Thyroid. 2013;23(6):683–94. Epub 2013/06/08. 10.1089/thy.2012.0307 . 23742290

[8] Kim TY, Kim WG, Kim WB, Shong YK. Current Status and Future Perspectives in Differentiated Thyroid Cancer. Endocrinol Metab. 2014;29(3):217–25. 10.3803/EnM.2014.29.3.217 PMC4192824 25309778

[9] Chung JK, Cheon GJ. Radioiodine Therapy in Differentiated Thyroid Cancer: The First Targeted Therapy in Oncology. Endocrinol Metab. 2014;29(3):233–9. 10.3803/EnM.2014.29.3.233 PMC4192819 25309780

[10] Davies L, Welch HG. Increasing incidence of thyroid cancer in the United States, 1973–2002. JAMA: the journal of the American Medical Association. 2006;295(18):2164–7. Epub 2006/05/11. 10.1001/jama.295.18.2164 . 16684987

[11] Ito Y, Kudo T, Takamura Y, Kobayashi K, Miya A, Miyauchi A. Lymph node recurrence in patients with N1b papillary thyroid carcinoma who underwent unilateral therapeutic modified radical neck dissection. World J Surg. 2012;36(3):593–7. 10.1007/s00268-011-1391-1. 10.1007/s00268-011-1391-1 22207493

[12] Ito Y, Kudo T, Kobayashi K, Miya A, Ichihara K, Miyauchi A. Prognostic factors for recurrence of papillary thyroid carcinoma in the lymph nodes, lung, and bone: analysis of 5,768 patients with average 10-year follow-up. World J Surg. 2012;36(6):1274–8. Epub 2012/01/25. 10.1007/s00268-012-1423-5 . 22270990

[13] Machens A, Dralle H. Correlation between the number of lymph node metastases and lung metastasis in papillary thyroid cancer. J Clin Endocrinol Metab. 2012;97(12):4375–82. 10.1210/jc.2012-1257 23019347

[14] Randolph GW, Duh QY, Heller KS, LiVolsi VA, Mandel SJ, Steward DL, et al The prognostic significance of nodal metastases from papillary thyroid carcinoma can be stratified based on the size and number of metastatic lymph nodes, as well as the presence of extranodal extension. Thyroid. 2012;22(11):1144–52. 10.1089/thy.2012.0043. 10.1089/thy.2012.0043 23083442

[15] Wang LY, Palmer FL, Nixon IJ, Tuttle RM, Shah JP, Patel SG, et al Lateral Neck Lymph Node Characteristics Prognostic of Outcome in Patients with Clinically Evident N1b Papillary Thyroid Cancer. Ann Surg Oncol. 2015 Epub 2015/02/11. 10.1245/s10434-015-4398-2 . 25665952 PMC4977989

[16] Lee YS, Lim YS, Lee JC, Wang SG, Kim IJ, Son SM, et al Clinical implications of bilateral lateral cervical lymph node metastasis in papillary thyroid cancer: a risk factor for lung metastasis. Ann Surg Oncol. 2011;18(12):3486–92. 10.1245/s10434-011-1763-7. 10.1245/s10434-011-1763-7 21553141

[17] Jeon MJ, Kim WG, Park WR, Han JM, Kim TY, Song DE, et al Modified dynamic risk stratification for predicting recurrence using the response to initial therapy in patients with differentiated thyroid carcinoma. European journal of endocrinology / European Federation of Endocrine Societies. 2014;170(1):23–30. Epub 2013/10/04. 10.1530/eje-13-0524 . 24088549

[18] Yim JH, Kim WB, Kim EY, Kim WG, Kim TY, Ryu JS, et al The outcomes of first reoperation for locoregionally recurrent/persistent papillary thyroid carcinoma in patients who initially underwent total thyroidectomy and remnant ablation. J Clin Endocrinol Metab. 2011;96(7):2049–56. Epub 2011/04/22. 10.1210/jc.2010-2298 . 21508143

[19] Jeon MJ, Yoon JH, Han JM, Yim JH, Hong SJ, Song DE, et al The prognostic value of the metastatic lymph node ratio and maximal metastatic tumor size in pathological N1a papillary thyroid carcinoma. European journal of endocrinology / European Federation of Endocrine Societies. 2013;168(2):219–25. 10.1530/EJE-12-0744 . 23161752

[20] Schlumberger M, Catargi B, Borget I, Deandreis D, Zerdoud S, Bridji B, et al Strategies of radioiodine ablation in patients with low-risk thyroid cancer. The New England journal of medicine. 2012;366(18):1663–73. Epub 2012/05/04. 10.1056/NEJMoa1108586 . 22551127

[21] Ferro DP, Falconi MA, Adam RL, Ortega MM, Lima CP, de Souza CA, et al Fractal characteristics of May-Grunwald-Giemsa stained chromatin are independent prognostic factors for survival in multiple myeloma. PloS one. 2011;6(6):e20706 Epub 2011/06/24. 10.1371/journal.pone.0020706 ; PubMed Central PMCID: PMCPmc3116829. 21698234 PMC3116829

[22] Reis-Alves SC, Traina F, Harada G, Campos PM, Saad ST, Metze K, et al Immunophenotyping in myelodysplastic syndromes can add prognostic information to well-established and new clinical scores. PloS one. 2013;8(12):e81048 Epub 2013/12/11. 10.1371/journal.pone.0081048 ; PubMed Central PMCID: PMCPmc3855682. 24324660 PMC3855682

[23] Metze K. Dichotomizing continuous prognostic factors can cause paradoxical results in survival models. Journal of the American College of Surgeons. 2011;212(1):132–4. Epub 2010/12/28. 10.1016/j.jamcollsurg.2010.10.004 . 21184962

[24] Kim SJ, Park SY, Lee YJ, Lee EK, Kim SK, Kim TH, et al Risk factors for recurrence after therapeutic lateral neck dissection for primary papillary thyroid cancer. Ann Surg Oncol. 2014;21(6):1884–90. Epub 2014/02/12. 10.1245/s10434-014-3507-y . 24515566

[25] Cooper DS, Doherty GM, Haugen BR, Kloos RT, Lee SL, Mandel SJ, et al Revised American Thyroid Association management guidelines for patients with thyroid nodules and differentiated thyroid cancer. Thyroid. 2009;19(11):1167–214. Epub 2009/10/29. 10.1089/thy.2009.0110 . 19860577

[26] Leboulleux S, Rubino C, Baudin E, Caillou B, Hartl DM, Bidart JM, et al Prognostic factors for persistent or recurrent disease of papillary thyroid carcinoma with neck lymph node metastases and/or tumor extension beyond the thyroid capsule at initial diagnosis. J Clin Endocrinol Metab. 2005;90(10):5723–9. Epub 2005/07/21. 10.1210/jc.2005-0285 . 16030160

[27] Ito Y, Fukushima M, Kihara M, Takamura Y, Kobayashi K, Miya A, et al Investigation of the prognosis of patients with papillary thyroid carcinoma by tumor size. Endocr J. 2012;59(6):457–64. . 22447137 10.1507/endocrj.ej12-0013

[28] Haymart MR. Understanding the relationship between age and thyroid cancer. The oncologist. 2009;14(3):216–21. Epub 2009/03/10. 10.1634/theoncologist.2008-0194 . 19270027

[29] Goffredo P, Sosa JA, Roman SA. Differentiated thyroid cancer presenting with distant metastases: a population analysis over two decades. World J Surg. 2013;37(7):1599–605. 10.1007/s00268-013-2006-9. 10.1007/s00268-013-2006-9 23525600

[30] Ibrahimpasic T, Nixon IJ, Palmer FL, Whitcher MM, Tuttle RM, Shaha A, et al Undetectable thyroglobulin after total thyroidectomy in patients with low- and intermediate-risk papillary thyroid cancer—is there a need for radioactive iodine therapy? Surgery. 2012;152(6):1096–105. 10.1016/j.surg.2012.08.034 23158181

[31] Byar DP, Green SB, Dor P, Williams ED, Colon J, van Gilse HA, et al A prognostic index for thyroid carcinoma. A study of the E.O.R.T.C. Thyroid Cancer Cooperative Group. European journal of cancer. 1979;15(8):1033–41. Epub 1979/08/01. . 510341 10.1016/0014-2964(79)90291-3

[32] Cady B, Rossi R. An expanded view of risk-group definition in differentiated thyroid carcinoma. Surgery. 1988;104(6):947–53. Epub 1988/12/01. . 3194846

[33] Kim SJ, Park SY, Lee YJ, Lee EK, Kim SK, Kim TH, et al Risk Factors for Recurrence After Therapeutic Lateral Neck Dissection for Primary Papillary Thyroid Cancer. Annals of surgical oncology. 2014 10.1245/s10434-014-3507-y. . 24515566

[34] Kim WG, Kim EY, Yim JH, Han JM, Jeon MJ, Kim TY, et al Comparison of Different Staging Systems for Predicting Recurrence of Papillary Thyroid Carcinoma. Endocrinol Metab. 2011;26(1):53–61. 10.3803/EnM.2011.26.1.53

